# Reducing pressure ulcers in patients with prolonged acute mechanical ventilation: a quasi-experimental study

**DOI:** 10.5935/0103-507X.20170007

**Published:** 2017

**Authors:** Cecilia Inés Loudet, María Cecilia Marchena, María Roxana Maradeo, Silvia Laura Fernández, María Victoria Romero, Graciela Esther Valenzuela, Isabel Eustaquia Herrera, Martha Teresa Ramírez, Silvia Rojas Palomino, Mariana Virginia Teberobsky, Leandro Ismael Tumino, Ana Laura González, Rosa Reina, Elisa Estenssoro

**Affiliations:** 1 Unidade de Terapia Intensiva, Hospital Interzonal General de Agudos "General San Martín" - La Plata, Buenos Aires, Argentina.; 2 Disciplina de Farmacologia Aplicada, Seção de Terapia Intensiva, Facultad de Ciencias Médicas, Universidad Nacional de La Plata - La Plata, Buenos Aires, Argentina.; 3 Serviço de Dermatologia, Hospital Interzonal General de Agudos "General San Martín" - La Plata, Buenos Aires, Argentina.

**Keywords:** Pressure ulcer/prevention & control, Respiration, artificial, Length of stay, Mobile applications/standards, Smartphone, Telemedicine/methods, Intensive care units

## Abstract

**Objective:**

To determine the effectiveness of a quality management program in reducing the incidence and severity of pressure ulcers in critical care patients.

**Methods:**

This was a quasi-experimental, before-and-after study that was conducted in a medical-surgical intensive care unit. Consecutive patients who had received mechanical ventilation for ≥ 96 hours were included. A "Process Improvement" team designed a multifaceted interventional process that consisted of an educational session, a pressure ulcer checklist, a smartphone application for lesion monitoring and decision-making, and a "family prevention bundle".

**Results:**

Fifty-five patients were included in Pre-I group, and 69 were included in the Post-I group, and the incidence of pressure ulcers in these groups was 41 (75%) and 37 (54%), respectively. The median time for pressure ulcers to develop was 4.5 [4 - 5] days in the Pre-I group and 9 [6 - 20] days in the Post-I group after admission for each period. The incidence of advanced-grade pressure ulcers was 27 (49%) in the Pre-I group and 7 (10%) in the Post-I group, and finally, the presence of pressure ulcers at discharge was 38 (69%) and 18 (26%), respectively (p < 0.05 for all comparisons). Family participation totaled 9% in the Pre-I group and increased to 57% in the Post-I group (p < 0.05). A logistic regression model was used to analyze the predictors of advanced-grade pressure ulcers. The duration of mechanical ventilation and the presence of organ failure were positively associated with the development of pressure ulcers, while the multifaceted intervention program acted as a protective factor.

**Conclusion:**

A quality program based on both a smartphone application and family participation can reduce the incidence and severity of pressure ulcers in patients on prolonged acute mechanical ventilation.

## Abstract (pt)

**Objetivo:**

Determinar a eficácia de um programa de gestão da qualidade para reduzir a incidência e a gravidade de úlceras por pressão em pacientes de terapia intensiva.

**Métodos:**

Estudo com metodologia quasi-experimental, antes-e-depois, conduzido em uma unidade de terapia intensiva clínica e cirúrgica. Incluíram-se os pacientes consecutivos que receberam ventilação mecânica por um período igual ou superior a 96 horas. Uma equipe de Melhoria de Processos delineou um processo de intervenção multifacetado, que consistiu de uma sessão educacional, uma lista de verificação de úlcera de pressão, um aplicativo para *smartphone* para monitoramento de lesões e um conjunto de normas de tomada de decisão, além de prevenção familiar.

**Resultados:**

O Grupo Pré-I incluiu 25 pacientes, e o Grupo Pós-I foi constituído por 69 pacientes. A incidência de úlcera de pressão nestes grupos foi de 41 (75%) e 37 (54%), respectivamente. O tempo mediano para o desenvolvimento das úlceras por pressão foi de 4,5 (4-5) dias no Grupo Pré-I e 9 (6-20) dias no Grupo Pós-I após a admissão para cada um dos períodos. A incidência de úlceras por pressão de grau avançado foi de 27 (49%) no Grupo Pré-I e 7 (10%) no Grupo Pós-I. A presença de úlceras por pressão na alta foi de 38 (69%) e 18 (26%), respectivamente, para os Grupos Pré-I e Pós-I (p < 0,05 para todas as comparações). A participação da família totalizou 9% no Grupo Pré-I e aumentou para 57% no Grupo Pós-I (p < 0,05). Utilizou-se um modelo de regressão logística para analisar os preditores de úlcera de pressão com grau avançado. A duração da ventilação mecânica e a presença de falência de órgão associaram-se positivamente com o desenvolvimento de úlceras por pressão, enquanto o programa multifacetado de intervenção atuou como fator de proteção.

**Conclusão:**

Um programa de qualidade, com base em um aplicativo para *smartphone* e na participação da família, pode reduzir a incidência e a gravidade de úlceras por pressão em pacientes com ventilação mecânica aguda prolongada.

## INTRODUCTION

Critical care patients are exposed to multiple problems related to the quality and safety of care.^(1)^ A frequent issue that these patients experience is the development of pressure ulcers (PUs), which are usually related to global and local hypoperfusion as well as exposure to excessive pressure, shearing forces, limited mobility, malnutrition, and other conditions. Pressure ulcers have also been associated with higher mortality and decreased quality of life.^(2,3)^ Therefore, the incidence and severity of PUs have become indicators of the quality of care and safety of patients in the intensive care unit (ICU).^(4)^

Prevalence and incidence studies indicate that PUs are common. Among different reports, prevalence rates range from 0.38% to 53.2%, and incidence can vary from 1.9% to 71.6% across Europe, Japan, China, the Middle East, the USA, Australia and Canada.^(5,6)^ The estimated incidence of PUs in acute care settings varies widely as well, from 3.3 to 53.4%.^(7)^

Few published studies have analyzed the incidence of PUs in Latin America, apart from Brazil, where there is a high incidence of PUs that have been reported in some regions. For example, one ICU in Brazil recorded an incidence of 53%; however, Brazil reports wide variability, with incidence varying from 5.8 to 55%.^(8)^

A recently published study conducted in our ICU between 2010 and 2012 aimed to describe the evolution of selected physical and psychological symptoms after discharge in ICU survivors who had received more than 48 hours of mechanical ventilation (MV) and detected serious issues regarding the incidence of PUs. At one month post-discharge, 75% of patients presented with PUs in addition to other physical consequences.^(9)^

To address this complication as part of a quality-of-care program, we designed a multifaceted intervention that focused on patients with prolonged acute MV (MV ≥ 96 hours).^(10)^ Our aim was to determine the effectiveness of this program in reducing the incidence and severity of PUs in this critical care population.

## METHODS

This was a quasi-experimental, before-and-after study that was conducted in a 14-bed medical-surgical ICU within a university-affiliated hospital. ICU patients who were adults (≥ 15 years old), who were consecutively enrolled and who required MV ≥ 96 hours were included in this study.^(10)^ Patients who had do-not-resuscitate orders and pre-existent PUs were not included in this study. The Ethical Review Board of the *Hospital San Martín de La Plata* approved this protocol (number: 001513; date: 01/01/13). Written, informed consent was obtained from relatives before the patients were included in the study.

This study consisted of a pre-intervention period of 7 months (Pre-I, June-December 2013) and a post-intervention period of 9 months (Post-I, April-December 2014); these periods were separated by the implementation of a multifaceted multidisciplinary intervention. During the Pre-I period, standard care was provided, which consisted of patient repositioning during every nursing shift (repositioning occurred only when the patients were hemodynamically stable, had normal intracranial pressure and had a closed abdomen) and use of hydrocolloid moisture-retentive wound dressings, heel floats and air mattresses. Thereafter, a 3-month "wash-in" phase ensued to allow time for full implementation of the protocol wherein standard PU care was maintained.

When developing the intervention, we first focused on the reality that there was only one dermatologist who specialized in soft tissue lesions and who was available for the entire hospital. Consequently, a "process improvement" task-force was formed to maximize the expertise of the specialist in an extremely limited time frame. The team was composed of 16 ICU nurses, 1 dermatologist, and 3 critical care specialists. Two physicians and 2 nurses were appointed as team leaders, had direct contact with the dermatologist and designed a multifaceted educational intervention.

**Figure 1:**
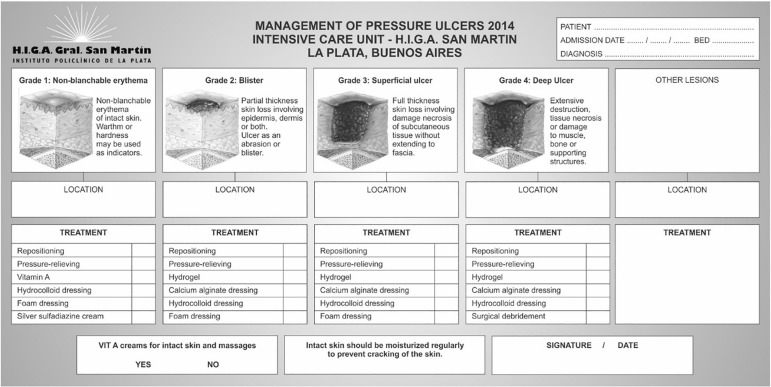
Form designed for monitoring and managing pressure ulcers.

Next, the ICU medical and nursing personnel were instructed by the dermatologist on lesion classification, wound cleansing methodology, and treatment indication as well as discussion of the different therapeutic options (i.e., the type of wound care product and the need for consultation with a surgical specialist) during four educational sessions. Thereafter, a daily head-to-toe inspection of the skin was performed, and upon completion of the inspection, a paper form for PU monitoring and treatment that was designed by the team was completed at the patient's bedside each time a change occurred or at least once during every 48-hour period (Figure 1).

Afterward, the use of the Whatsapp^®^ smartphone application was implemented to monitor and communicate therapeutic decisions concerning PUs on a daily basis. For this task, 2 groups were formed to streamline communication. The main group who made decisions regarding patient care included team leaders and the dermatologist, and this group conducted systematic evaluations, which included photographing the lesions, making sure not to include any identifying patient features. The second, larger group, which followed the instructions provided by the main group, included the entire nursing staff and received all the photographs and staging and management instructions but did not consult directly with the dermatologist due to the size of the nursing pool. All staff members were educated on the ethical considerations of using photographs for educational and therapeutic purposes, and great care was taken to ensure that no identifying patient features were included in the photographs.

Finally, the last component added to the intervention was the involvement of the patient's family. After the family received training from the staff, family members who volunteered to participate in the "family prevention bundle" agreed to perform pre-specified, limited activities for a minimum of two hours per day, twice a day, seven days a week. The bundle included daily monitoring of skin for the detection of new skin lesions and for the evolution of older lesions, daily application of lotions and vitamin A creams for hydration or silicone sprays for bony prominences, and assisting in rotating the patient together with the nursing staff. Most families took advantage of the open visitation policy, were present throughout the day and enthusiastically conducted their bundle duties.

In each period, we recorded the epidemiological variables, the reasons for admission, the severity-of-illness score on admission using Acute Physiology and Chronic Health Evaluation - APACHE-II and Sequential Organ Failure Assessment on admission - SOFA_24_ scores, the duration of mechanical ventilation and the length of the ICU stay as well as the in-hospital mortality, and the nurse:patient ratio.^(11,12)^ The outcome measures were calculated as indicators for the prevention and treatment of PUs. The risk of developing PUs was evaluated using the Braden Scale. The scale ranges from 6 to 23 points: grade 0, without risk (19 to 23 points); grade 1, mild risk (15 to 18 points); grade 2, moderate risk (13 to 14 points); grade 3, high risk (10 to 12 points); and grade 4, severe risk (6 to 9 points).^(13)^ This scale was selected because it is one of the most widely used scales in the critical care arena. Other outcome measurements that were calculated included the rate of patients using pressure-prevention mattresses, the incidence and location of PUs as well as the number of days it took to develop them, the number of PUs per patient, the incidence of advanced-grade PUs (3 or 4),^(14)^ the rate of patients with PUs at the time of ICU discharge, and the rate of family participation.

### Statistical analysis

The data are presented as percentages, mean ± standard deviation (SD), or median and interquartile ranges (IQR 25 - 75%). Comparisons were made between the Pre-I and Post-I groups. Continuous variables were compared using *t*-tests or the Mann-Whitney U test, according to their distribution, and categorical variables were compared using the chi-square test.

A logistic regression analysis was conducted to identify the independent variables that were related to the development of advanced-grade PUs. Predetermined variables, and those that were significantly associated with advanced-grade PUs in the univariate analysis (p < 0.20) were included in the multivariate analysis. The model was calibrated using the Hosmer-Lemeshow test; discrimination, using the receiver operating characteristic (ROC) curve. For all comparisons, a p-value of ≤ 0.05 was considered statistically significant. All analyses were performed with STATA 11.1 software. The SQUIRE 2.0 guideline was used for quality improvement reporting.^(15)^ The sample size was calculated after taking a baseline PU incidence of 75% into consideration.^(9)^ Anticipating a Post-I reduction in PUs of at least 45%, a two-sided α of 0.05 and a power of 80%, the number of patients required per period was ≥ 48. After adding 20% for possible losses, the final total N required was ≥ 116.

## RESULTS

**Figure 2:**
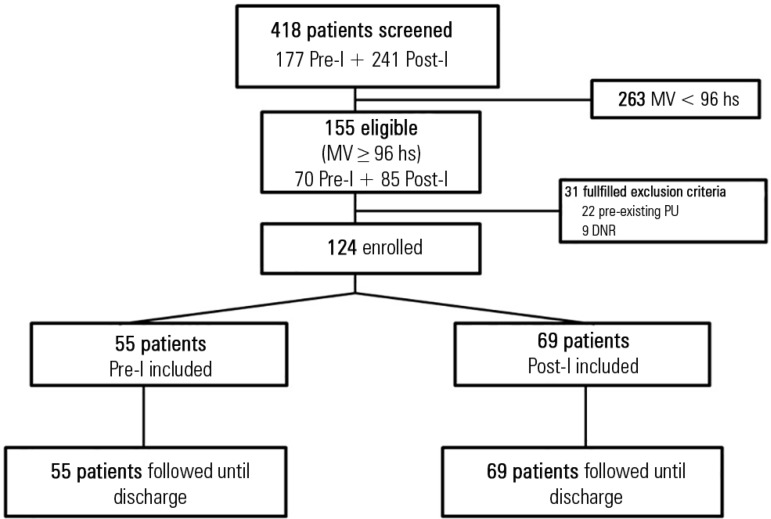
Flow-chart of the study. MV - mechanical ventilation; PU - pressure ulcers; DNR - do-not-resuscitate orders.

Of 418 patients who were admitted to the ICU during the study period, 263 were not included because the time that they spent on MV was < 96 hours. Of 155 eligible patients (70 in the Pre-I group; 85 in the Post-I group), an additional 31 patients were excluded (22 had pre-existing PUs, and 9 had do-not-resuscitate orders). Consequently, 124 patients met the inclusion criteria of MV ≥ 96 hours and an absence of previous lesions; 55 patients were enrolled in the Pre-I period, and 69 patients were enrolled in the Post-I period (Figure 2). Relevant patient characteristics for both periods are shown in table 1. Briefly, this population was young and acutely ill; the mean patient age was higher in the Post-I period. Medical diagnoses and severe organ failure predominated, and both multiple trauma and acute brain injury were frequent causes of admission. These patients exhibited a prolonged duration of mechanical ventilation and length of ICU stay, and the mortality was high. The nurse:patient ratio remained unchanged throughout the study in both periods.

**Table 1:** Characteristics of patients in pre- and post-intervention periods

Variables	Pre-intervention period	Post-intervention period	p value
Number of patients	55	69	
Age (years)	47 ± 18	39 ± 17	0.01
APACHE II score	18 ± 7	18 ± 6	0.77
SOFA_24_ score	7 [4 - 9]	8 [6 - 10]	0.06
Medical admission	31 (56)	33 (48)	0.35
Multiple trauma	6 (10)	14 (20)	0.13
Traumatic brain injury	8 (15)	13 (19)	0.51
Shock on admission	26 (48)	42 (63)	0.11
Dialysis	9 (17)	6 (8)	0.16
MV duration (days)	18 [9 - 46]	14 [8 - 34]	0.55
ICU LOS (days)	23 [8 - 47]	19.5 [11 - 36]	0.98
ICU mortality	23 (42)	24 (35)	0.42
Nurse:patient ratio	1: 2.5	1: 2.4	0.86

APACHE - Acute Physiology and Chronic Health Evaluation; SOFA24 - Sequential Organ Failure Assessment on admission; LOS - length of stay; MV - mechanical ventilation; ICU - intensive care unit. The data are presented as N (%), mean ± standard deviation or median [p25 - 75].

Performance indicators for the prevention and treatment of PUs in both periods are detailed in table 2. The risk of developing PUs according to the Braden Scale was similar in both periods. However, in the Post-I period, the use of pressure-prevention mattresses increased from 26 (48%) to 59 (85%) (p = 0.0000), and the rate of family participation improved from 5 (9%) to 39 (57%) (p = 0.0000). The global pressure ulcer incidence decreased in the Post-I period from 41 (75%) to 37 (54%) (p = 0.016). There was a notable decrease in advanced-grade PUs from 27 (49%) to 7 (10%) (p = 0.0000). The sacral, heel and other PU locations were also significantly reduced in the Post-I period. In addition, the onset of new lesions was delayed from a median of 4.5 [4 - 5] to 9 [6 - 20] days (p = 0.0001), and lesions that were present at ICU discharge decreased from 38 (69%) to 18 (26%) (p = 0.0000).

**Table 2:** Indicators for the prevention and treatment of pressure ulcers

Indicators related to the prevention and treatment of PUs	Pre-intervention period	Post-intervention period	p value
Number of patients	55	69	
High or severe PU risk development (Braden Scale*)	50 (91)	62 (90)	0.84
Braden Score* grades	3 [3 - 4]	3 [3 - 4]	0.87
Use of pressure prevention mattresses	26 (48)	59 (85)	0.0000
Family participation	5 (9)	39 (57)	0.0000
Pressure ulcer incidence	41 (75)	37 (54)	0.016
Number of PU/patient	2.10 ± 1.10	1.02 ± 0.42	0.0000
Advanced-grade PU (grades 3 and 4)	27 (49)	7 (10)	0.0000
Advanced-grade PU, N°/total N° PU (%)	27/41 (66)	7/37 (19)	0.0000
PU location†			
Sacral	37 (67)	30 (43)	0.0083
Heels	39 (71)	24 (35)	0.0001
Other	16 (29)	8 (12)	0.014
Days to develop PU	4.5 [4 - 5]	9 [6 - 20]	0.0001
Pressure ulcer at discharge	38 (69)	18 (26)	0.0000

PU - pressure ulcer.

* Pressure ulcer risk development: 1: mild; 2: moderate; 3: high; 4: severe.

† Patients can have pressure ulcers in more than one location. The data are presented as N (%), mean ± standard deviation or median [p25-75].

The logistic regression model indicated that the MV duration and SOFA_24_ score were positively associated with advanced-grade PUs, while the multifaceted educational intervention acted as a protective factor (Table 3). Adjustment and discrimination of the model were appropriate. The Hosmer-Lemeshow test was 3.71 (p = 0.86), and the area below the ROC curve was 0.88 (95%CI: 0.81 - 0.96).

**Table 3:** Multivariate logistic regression model for advanced-grade pressure ulcers

Advanced-grade PU	Odds ratio	SE	p value	95% Confidence interval
MV duration* (d)	1.04	0.015	0.006	1.012 - 1.070
SOFA_24_ *	1.43	0.166	0.002	1.140 - 1.798
Multifaceted intervention	0.04	0.031	0.000	0.009 - 0.186

PU - pressure ulcers; SE - standard error; MV - mechanical ventilation; SOFA_24_ - Sequential Organ Failure Assessment at admission;

* The odds ratio increased with respect to the units of mechanical ventilation or SOFA_24_ score.

## DISCUSSION

This study demonstrated that a multifaceted educational intervention that consisted of the implementation of a multidisciplinary team for the management of PUs, a smartphone application as a telemedicine tool for lesion oversight, and the participation of families in patient care decreased the incidence and severity of lesions. Furthermore, we found that SOFA_24_ score and mechanical ventilation duration - both markers of acute, severe disease - were independent risk factors for the occurrence of advanced-grade pressure ulcers. Notably, the multifaceted educational intervention acted as the only protective predictor. Additionally, the onset of lesions was significantly delayed in the Post-I period, and the percentage of patients discharged without lesions was higher.

The risk of developing PUs in our ICU is high. Taking into consideration the entire population screened during the study period, the total incidence of PUs for all ICU patients was 23% in the Pre-I period and 15% in the Post-I period (data not presented). Considering only patients with MV ≥ 96 hours, the incidence of PUs was even higher (75% in the Pre-I period and 54% in the Post-I period); these patients were at the highest risk given their high grades (3 and 4) on the Braden Scale, prolonged MV duration, and immobilization due to shock, as well as the high incidence of multiple trauma and traumatic brain injury. Considering this scenario, no matter how comprehensive the protocol is that we design, PUs will still develop in some patients. Therefore, our multifaceted approach had the ultimate goal of reducing not only the global incidence of PUs, but also the number of advanced-grade lesions; we succeeded in this latter goal, with the incidence decreasing from 49% to 10%.

Another issue that complicated the high incidence of PUs is that the possibility of discharge to tertiary care institutions in Argentina - as well as Latin America in general - is limited; therefore, patients may remain in the ICU for months. Furthermore, the nurse:patient ratio in our ICU is clearly insufficient (1:2.4 - 2.5), with values similar to those in Latin America, (1:1.8 [1.0 - 2.6]), which are some of the lowest in the world.^(16)^ Insufficient clinical care staff is a well-known predictor of adverse outcomes.^(17)^

For evaluating and improving quality-of-care, the "monitoring system" approach focuses on the performance and periodic evaluation of selected indicators, while the PDSA (*Plan, Do, Study, Act*) cycle first identifies a problem, analyses it, and finally, proposes improvements^(18)^ to respond to the question "What can we improve?"^(19)^ Our study combined both approaches and commenced with identifying the problem, applying the improvement strategy, and finally, establishing monitoring guidelines.^(20)^

It is generally accepted that multicomponent interventions might be more effective than any individual approach for the prevention of PUs (e.g., the use of devices for pressure relief, such as advanced static mattresses or static overlays).^(6,21,22)^ In a recent ICU study in the US, a multifaceted prevention program reduced the incidence of PUs from 10% to 3%.^(7)^ Many other studies that have attempted to decrease the development of PUs only used singular interventions. For example, the *turn team* proposed in a study by Still et al. reduced lesions by turning patients every two hours.^(23)^ In the Behrendt et al. study, PUs were reduced by continuous bedside pressure mapping.^(24)^

Some elements of our approach have been used in limited degrees by other researchers. For example, in the de Araujo et al. study, the authors used digital photography over three months to classify lesions; however, of the 42 patients who participated in the study, only 47 grade 1 and 2 lesions were identified.^(25)^ Our study catalogued more than 1,500 photographs over a 16-month period and recorded all four grades of PUs. We maintained the standard practices for care of PUs as indicated above but also incorporated other elements, such as the smartphone application and the family prevention bundle. To our knowledge, this is the first study to incorporate this combination of different approaches.

Incorporating WhatsApp^®^ enabled the team to maximize the limited time of the specialist by focusing on the most severe lesions. Photographs of the lesions were simultaneously sent to all staff members, which allowed for timely monitoring and instantaneous comparison with the prior state of the lesion. The sheer number of photographs that were evaluated during the study dramatically increased the less-trained staff's exposure to the evaluation and treatment of PUs since not all personnel had the same knowledge on the prevention and treatment of this complication. This discrepancy in knowledge has also been noted in other studies.^(26)^

Family participation is a controversial issue for critical care staff. Most studies have explored the intention of family members to involve themselves in patient care, but few have reported active participation in specific tasks without overstepping boundaries with the staff, as we have successfully illustrated in the present study.^(27-29)^ It has been well documented that the presence of family aids in patient recovery.^(30,31)^ In two previous studies, family members expressed that massages were one of the main elements of healthcare they could most readily provide to increase a feeling of mutual well-being.^(32,33)^ Thus, we harnessed the curative aspects of the family presence and then added specific, yet limited, tasks that family members could provide while reducing the burden on the limited nursing staff. This engagement was possible due to the open visitation policy, which allowed family members to be present for extended periods.^(34)^

The findings of a recent American College of Critical Care Medicine Task Force on Models of Critical Care also supported many of our conclusions: (1) an intensivist-led, high-performing, multidisciplinary team dedicated to the ICU is an integral part of effective care delivery; (2) process improvement is the cornerstone of achieving high-quality ICU outcomes; and (3) standardized protocols including care bundles and protocols to facilitate measurable processes and outcomes should be used and further developed in the ICU setting.^(35)^

A limitation of this study was that it was conducted in only one public center, which compromises its external validity; however, the simplicity of the intervention allows for eventual generalization. Age was also a limitation in that this was a relatively young population (47 *versus* 39); therefore, we cannot completely rule out age as a predictor. However, in our model, age was not independently associated with more advanced-grade PUs. Another limitation was that we only evaluated family participation and not family satisfaction. As none of the family members refused to participate or quit, our impression was that their feeling of usefulness increased their involvement in their loved one's recovery. Finally, we cannot discard that awareness of good clinical practices by nurses could have contributed to better clinical outcomes, as they knew they were being observed (Hawthorne effect), regardless of any intervention. However, this is a collateral benefit that has been frequently described in before-after quality studies.

The main strength of this study is the possibility of generalization to any hospital setting, no matter the available resources. For example, in hospitals with generous nurse:patient ratios and support staff, educating family members on specific tasks such as the application of lotions or creams can offer them a feeling of usefulness in a situation in which they might otherwise feel helpless. In contrast, in hospitals with limited staff, incorporating family members in controlled tasks can serve as an invaluable resource. Another strength of the study is that there was no monetary cost or increase in staff associated with the implementation of the intervention. Of course, time, education and organizational costs applied, but these are inherent to all hospital settings, and these costs were negligible. Associated with cost is the idea presented in the previously mentioned US study that highlights the overall cost-savings for the hospital through the implementation of this kind of intervention.^(7)^

## CONCLUSION

It was feasible to significantly reduce the incidence and the severity of pressure ulcers in a high-risk population through the implementation of a multifaceted educational intervention that included the voluntary participation of a patient's family members. A no-cost smartphone application was utilized to reach this goal in combination with free educational components for personnel.
